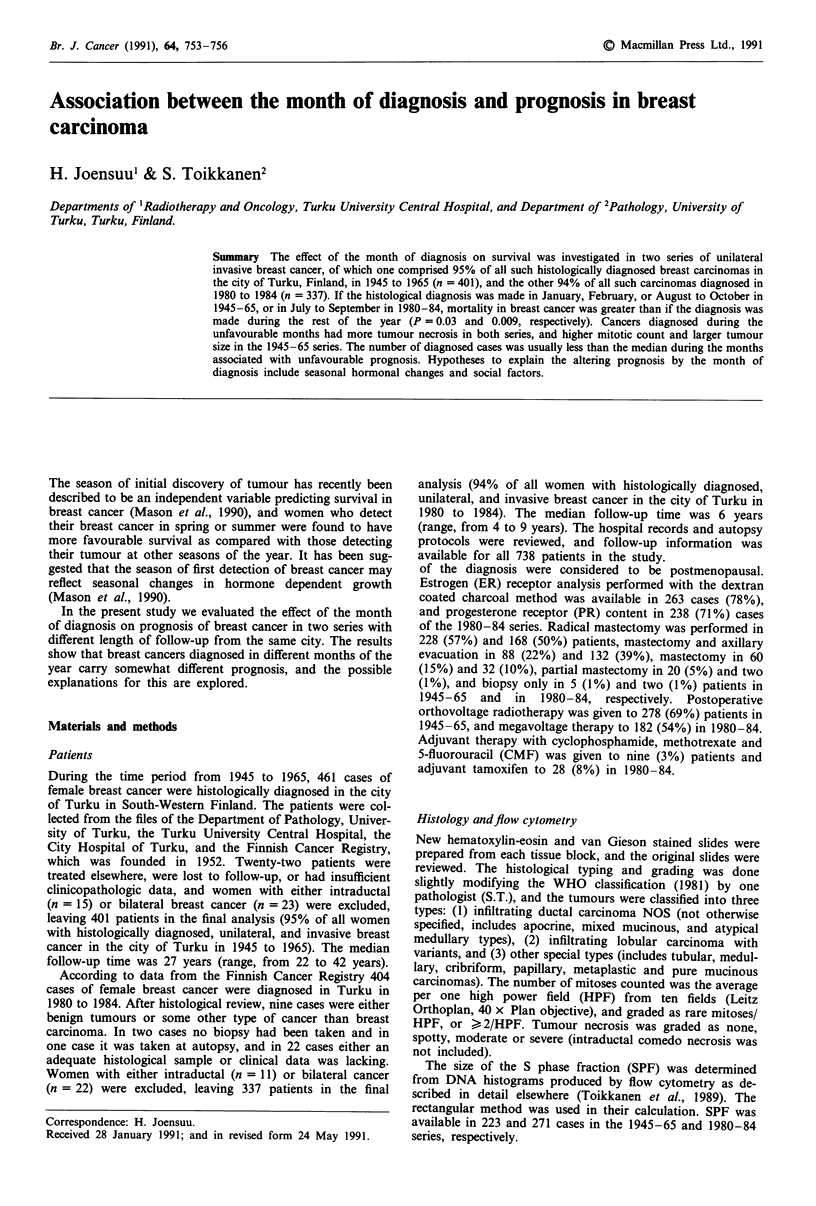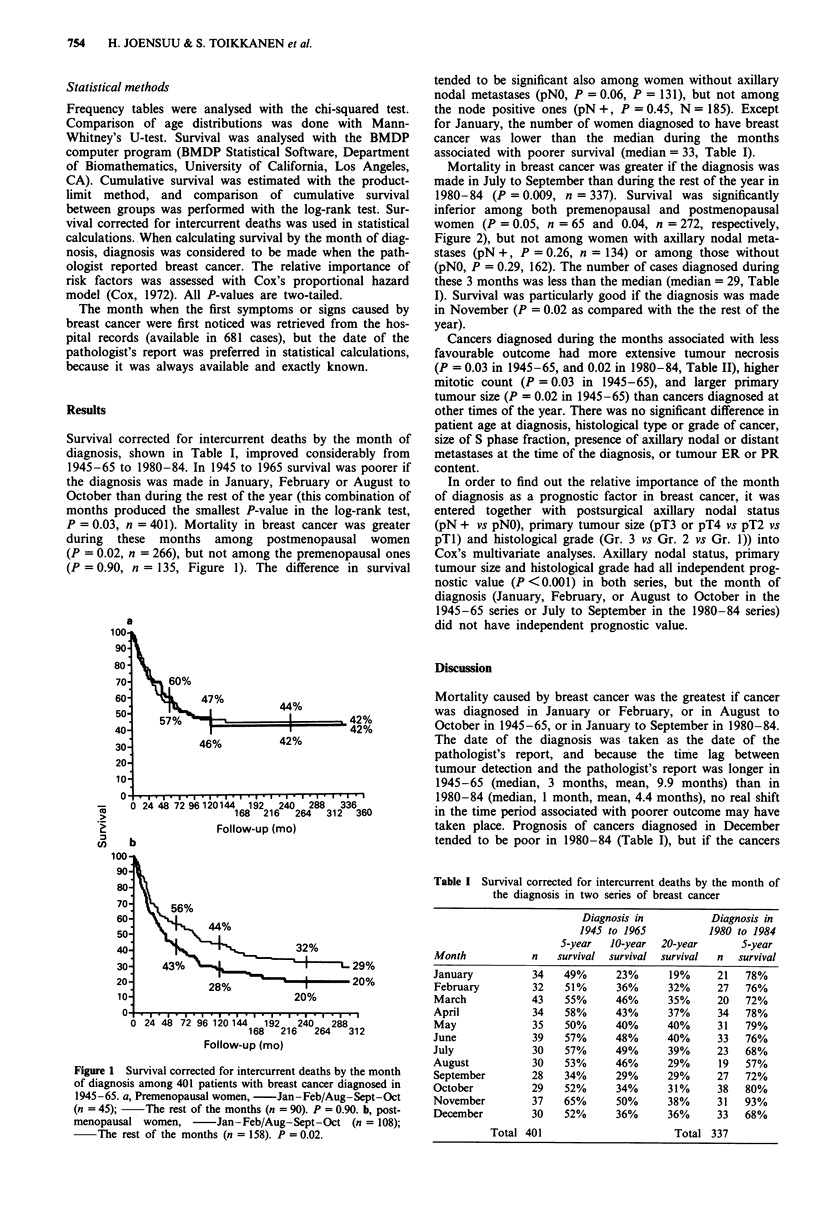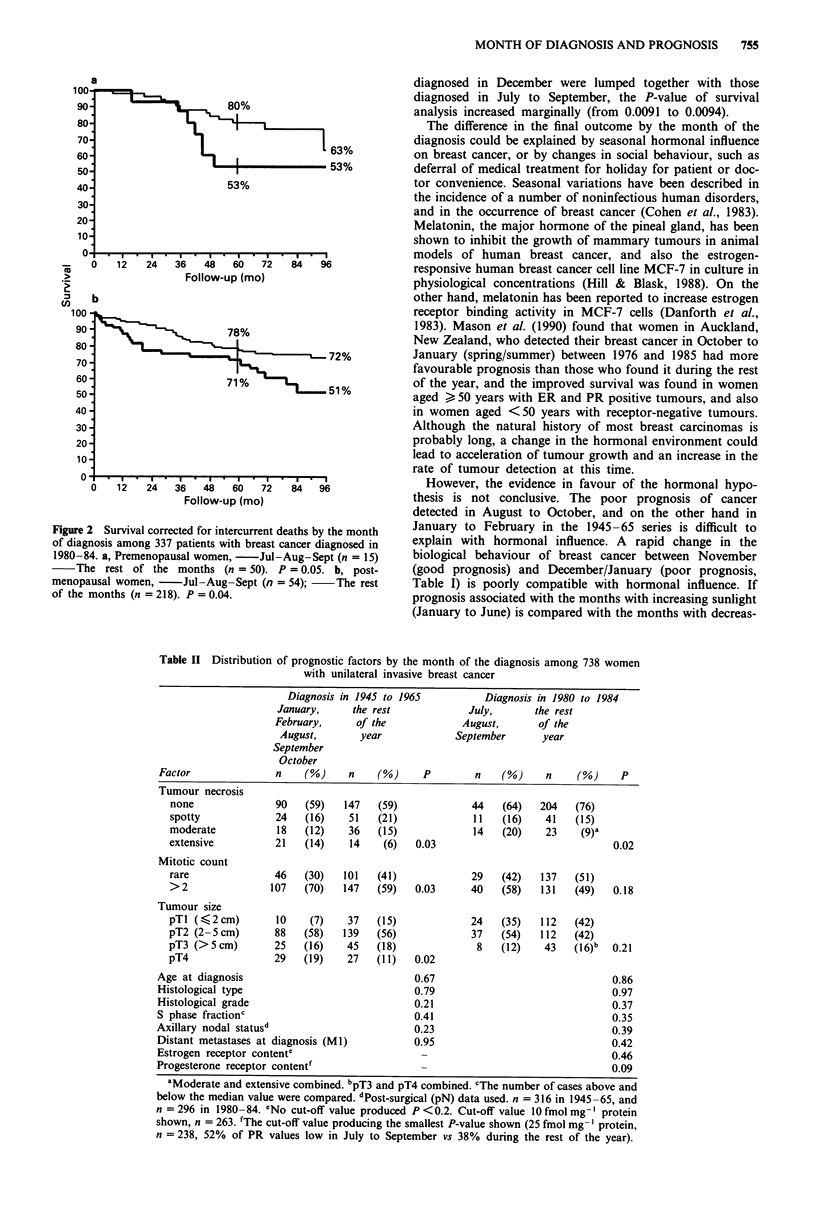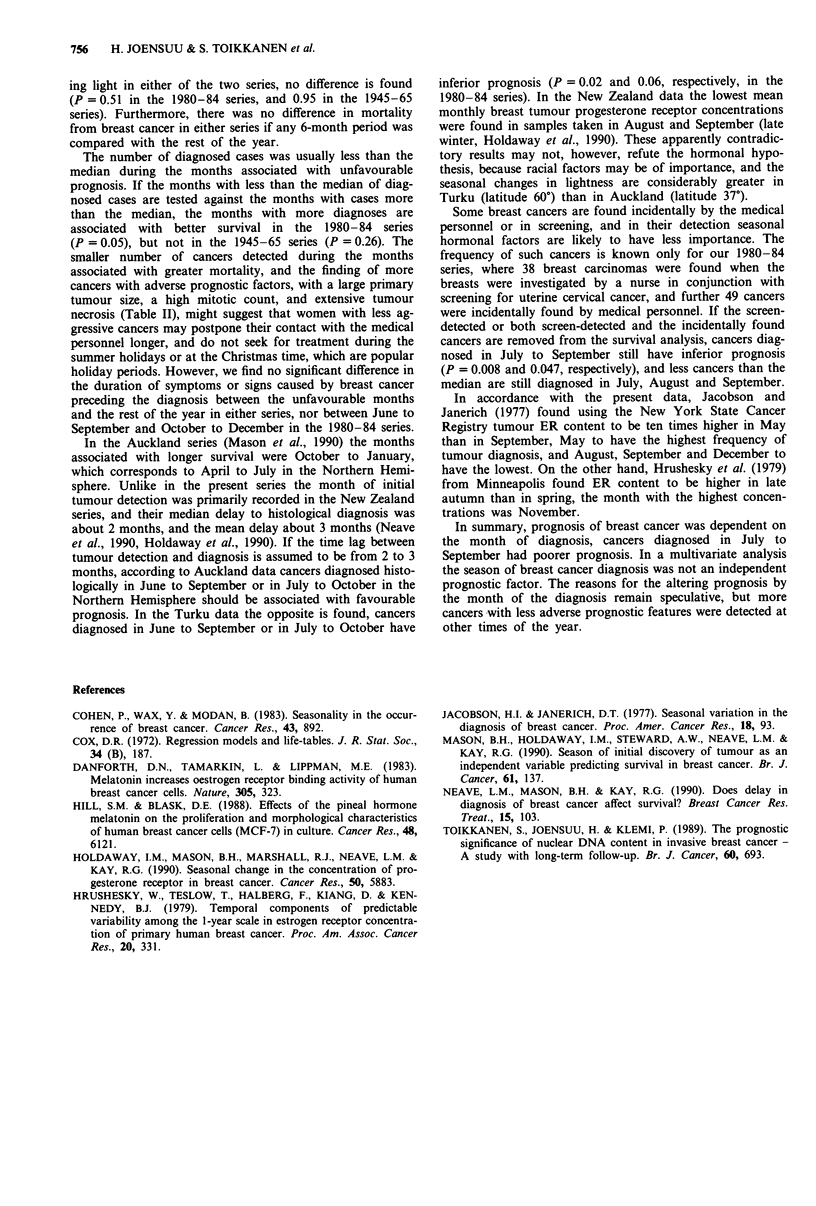# Association between the month of diagnosis and prognosis in breast carcinoma.

**DOI:** 10.1038/bjc.1991.393

**Published:** 1991-10

**Authors:** H. Joensuu, S. Toikkanen

**Affiliations:** Department of Radiotherapy, Turku University Central Hospital, Finland.

## Abstract

The effect of the month of diagnosis on survival was investigated in two series of unilateral invasive breast cancer, of which one comprised 95% of all such histologically diagnosed breast carcinomas in the city of Turku, Finland, in 1945 to 1965 (n = 401), and the other 94% of all such carcinomas diagnosed in 1980 to 1984 (n = 337). If the histological diagnosis was made in January, February, or August to October in 1945-65, or in July to September in 1980-84, mortality in breast cancer was greater than if the diagnosis was made during the rest of the year (P = 0.03 and 0.009, respectively). Cancers diagnosed during the unfavourable months had more tumour necrosis in both series, and higher mitotic count and larger tumour size in the 1945-65 series. The number of diagnosed cases was usually less than the median during the months associated with unfavourable prognosis. Hypotheses to explain the altering prognosis by the month of diagnosis include seasonal hormonal changes and social factors.


Br. J. Cancer (1991), 64, 753-756 © Macmillan Press Ltd., 1991

### Association between the month of diagnosis and prognosis in breast carcinoma

H. Joensuu[1] & S. Toikkanen[2]

*Departments of [1]Radiotherapy and Oncology, Turku University Central Hospital, and Department of [2]Pathology, University of Turku, Turku, Finland.*

**Summary** The effect of the month of diagnosis on survival was investigated in two series of unilateral invasive breast cancer, of which one comprised 95% of all such histologically diagnosed breast carcinomas in the city of Turku, Finland, in 1945 to 1965 ($n$ = 401), and the other 94% of all such carcinomas diagnosed in 1980 to 1984 ($n$ = 337). If the histological diagnosis was made in January, February, or August to October in 1945-65, or in July to September in 1980-84, mortality in breast cancer was greater than if the diagnosis was made during the rest of the year ($P$ = 0.03 and 0.009, respectively). Cancers diagnosed during the unfavourable months had more tumour necrosis in both series, and higher mitotic count and larger tumour size in the 1945-65 series. The number of diagnosed cases was usually less than the median during the months associated with unfavourable prognosis. Hypotheses to explain the altering prognosis by the month of diagnosis include seasonal hormonal changes and social factors.

The season of initial discovery of tumour has recently been described to be an independent variable predicting survival in breast cancer (Mason *et al.*, 1990), and women who detect their breast cancer in spring or summer were found to have more favourable survival as compared with those detecting their tumour at other seasons of the year. It has been suggested that the season of first detection of breast cancer may reflect seasonal changes in hormone dependent growth (Mason *et al.*, 1990).

In the present study we evaluated the effect of the month of diagnosis on prognosis of breast cancer in two series with different length of follow-up from the same city. The results show that breast cancers diagnosed in different months of the year carry somewhat different prognosis, and the possible explanations for this are explored.

### Materials and methods

### *Patients*

During the time period from 1945 to 1965, 461 cases of female breast cancer were histologically diagnosed in the city of Turku in South-Western Finland. The patients were collected from the files of the Department of Pathology, University of Turku, the Turku University Central Hospital, the City Hospital of Turku, and the Finnish Cancer Registry, which was founded in 1952. Twenty-two patients were treated elsewhere, were lost to follow-up, or had insufficient clinicopathologic data, and women with either intraductal ($n$ = 15) or bilateral breast cancer ($n$ = 23) were excluded, leaving 401 patients in the final analysis (95% of all women with histologically diagnosed, unilateral, and invasive breast cancer in the city of Turku in 1945 to 1965). The median follow-up time was 27 years (range, from 22 to 42 years).

According to data from the Finnish Cancer Registry 404 cases of female breast cancer were diagnosed in Turku in 1980 to 1984. After histological review, nine cases were either benign tumours or some other type of cancer than breast carcinoma. In two cases no biopsy had been taken and in one case it was taken at autopsy, and in 22 cases either an adequate histological sample or clinical data was lacking. Women with either intraductal ($n$ = 11) or bilateral cancer ($n$ = 22) were excluded, leaving 337 patients in the final analysis (94% of all women with histologically diagnosed, unilateral, and invasive breast cancer in the city of Turku in 1980 to 1984). The median follow-up time was 6 years (range, from 4 to 9 years). The hospital records and autopsy protocols were reviewed, and follow-up information was available for all 738 patients in the study.

of the diagnosis were considered to be postmenopausal. Estrogen (ER) receptor analysis performed with the dextran coated charcoal method was available in 263 cases (78%), and progesterone receptor (PR) content in 238 (71%) cases of the 1980-84 series. Radical mastectomy was performed in 228 (57%) and 168 (50%) patients, mastectomy and axillary evacuation in 88 (22%) and 132 (39%), mastectomy in 60 (15%) and 32 (10%), partial mastectomy in 20 (5%) and two (1%), and biopsy only in 5 (1%) and two (1%) patients in 1945-65 and in 1980-84, respectively. Postoperative orthovoltage radiotherapy was given to 278 (69%) patients in 1945-65, and megavoltage therapy to 182 (54%) in 1980-84. Adjuvant therapy with cyclophosphamide, methotrexate and 5-fluorouracil (CMF) was given to nine (3%) patients and adjuvant tamoxifen to 28 (8%) in 1980-84.

### *Histology and flow cytometry*

New hematoxylin-eosin and van Gieson stained slides were prepared from each tissue block, and the original slides were reviewed. The histological typing and grading was done slightly modifying the WHO classification (1981) by one pathologist (S.T.), and the tumours were classified into three types: (1) infiltrating ductal carcinoma NOS (not otherwise specified, includes apocrine, mixed mucinous, and atypical medullary types), (2) infiltrating lobular carcinoma with variants, and (3) other special types (includes tubular, medullary, cribriform, papillary, metaplastic and pure mucinous carcinomas). The number of mitoses counted was the average per one high power field (HPF) from ten fields (Leitz Orthoplan, 40 × Plan objective), and graded as rare mitoses/HPF, or ⩾2/HPF. Tumour necrosis was graded as none, spotty, moderate or severe (intraductal comedo necrosis was not included).

The size of the S phase fraction (SPF) was determined from DNA histograms produced by flow cytometry as described in detail elsewhere (Toikkanen *et al.*, 1989). The rectangular method was used in their calculation. SPF was available in 223 and 271 cases in the 1945-65 and 1980-84 series, respectively.

Correspondence: H. Joensuu.
Received 28 January 1991; and in revised form 24 May 1991.

*Statistical methods*

Frequency tables were analysed with the chi-squared test. Comparison of age distributions was done with Mann-Whitney's U-test. Survival was analysed with the BMDP computer program (BMDP Statistical Software, Department of Biomathematics, University of California, Los Angeles, CA). Cumulative survival was estimated with the product-limit method, and comparison of cumulative survival between groups was performed with the log-rank test. Survival corrected for intercurrent deaths was used in statistical calculations. When calculating survival by the month of diagnosis, diagnosis was considered to be made when the pathologist reported breast cancer. The relative importance of risk factors was assessed with Cox's proportional hazard model (Cox, 1972). All *P*-values are two-tailed.

The month when the first symptoms or signs caused by breast cancer were first noticed was retrieved from the hospital records (available in 681 cases), but the date of the pathologist's report was preferred in statistical calculations, because it was always available and exactly known.

### Results

Survival corrected for intercurrent deaths by the month of diagnosis, shown in Table I, improved considerably from 1945–65 to 1980–84. In 1945 to 1965 survival was poorer if the diagnosis was made in January, February or August to October than during the rest of the year (this combination of months produced the smallest *P*-value in the log-rank test, $P = 0.03$, $n = 401$). Mortality in breast cancer was greater during these months among postmenopausal women ($P = 0.02$, $n = 266$), but not among the premenopausal ones ($P = 0.90$, $n = 135$, Figure 1). The difference in survival

**Figure 1** Survival corrected for intercurrent deaths by the month of diagnosis among 401 patients with breast cancer diagnosed in 1945–65. *a*, Premenopausal women, ——Jan–Feb/Aug–Sept–Oct ($n = 45$); ——The rest of the months ($n = 90$). $P = 0.90$. **b**, postmenopausal women, ——Jan–Feb/Aug–Sept–Oct ($n = 108$); ——The rest of the months ($n = 158$). $P = 0.02$.

tended to be significant also among women without axillary nodal metastases (pN0, $P = 0.06$, $P = 131$), but not among the node positive ones (pN +, $P = 0.45$, N = 185). Except for January, the number of women diagnosed to have breast cancer was lower than the median during the months associated with poorer survival (median = 33, Table I).

Mortality in breast cancer was greater if the diagnosis was made in July to September than during the rest of the year in 1980–84 ($P = 0.009$, $n = 337$). Survival was significantly inferior among both premenopausal and postmenopausal women ($P = 0.05$, $n = 65$ and 0.04, $n = 272$, respectively, Figure 2), but not among women with axillary nodal metastases (pN +, $P = 0.26$, $n = 134$) or among those without (pN0, $P = 0.29$, 162). The number of cases diagnosed during these 3 months was less than the median (median = 29, Table I). Survival was particularly good if the diagnosis was made in November ($P = 0.02$ as compared with the the rest of the year).

Cancers diagnosed during the months associated with less favourable outcome had more extensive tumour necrosis ($P = 0.03$ in 1945–65, and 0.02 in 1980–84, Table II), higher mitotic count ($P = 0.03$ in 1945–65), and larger primary tumour size ($P = 0.02$ in 1945–65) than cancers diagnosed at other times of the year. There was no significant difference in patient age at diagnosis, histological type or grade of cancer, size of S phase fraction, presence of axillary nodal or distant metastases at the time of the diagnosis, or tumour ER or PR content.

In order to find out the relative importance of the month of diagnosis as a prognostic factor in breast cancer, it was entered together with postsurgical axillary nodal status (pN + *vs* pN0), primary tumour size (pT3 or pT4 *vs* pT2 *vs* pT1) and histological grade (Gr. 3 *vs* Gr. 2 *vs* Gr. 1)) into Cox's multivariate analyses. Axillary nodal status, primary tumour size and histological grade had all independent prognostic value ($P < 0.001$) in both series, but the month of diagnosis (January, February, or August to October in the 1945–65 series or July to September in the 1980–84 series) did not have independent prognostic value.

### Discussion

Mortality caused by breast cancer was the greatest if cancer was diagnosed in January or February, or in August to October in 1945–65, or in January to September in 1980–84. The date of the diagnosis was taken as the date of the pathologist's report, and because the time lag between tumour detection and the pathologist's report was longer in 1945–65 (median, 3 months, mean, 9.9 months) than in 1980–84 (median, 1 month, mean, 4.4 months), no real shift in the time period associated with poorer outcome may have taken place. Prognosis of cancers diagnosed in December tended to be poor in 1980–84 (Table I), but if the cancers

**Table I** Survival corrected for intercurrent deaths by the month of the diagnosis in two series of breast cancer

| Month | *Diagnosis in 1945 to 1965* *n* | *5-year survival* | *10-year survival* | *20-year survival* | *Diagnosis in 1980 to 1984* *n* | *5-year survival* |
|---|---|---|---|---|---|---|
| January | 34 | 49% | 23% | 19% | 21 | 78% |
| February | 32 | 51% | 36% | 32% | 27 | 76% |
| March | 43 | 55% | 46% | 35% | 20 | 72% |
| April | 34 | 58% | 43% | 37% | 34 | 78% |
| May | 35 | 50% | 40% | 40% | 31 | 79% |
| June | 39 | 57% | 48% | 40% | 33 | 76% |
| July | 30 | 57% | 49% | 39% | 23 | 68% |
| August | 30 | 53% | 46% | 29% | 19 | 57% |
| September | 28 | 34% | 29% | 29% | 27 | 72% |
| October | 29 | 52% | 34% | 31% | 38 | 80% |
| November | 37 | 65% | 50% | 38% | 31 | 93% |
| December | 30 | 52% | 36% | 36% | 33 | 68% |
| Total | 401 | | | Total | 337 | |

**Figure 2** Survival corrected for intercurrent deaths by the month of diagnosis among 337 patients with breast cancer diagnosed in 1980–84. **a**, Premenopausal women, ——Jul–Aug–Sept ($n$ = 15) ——The rest of the months ($n$ = 50). $P$ = 0.05. **b**, post-menopausal women, ——Jul–Aug–Sept ($n$ = 54); ——The rest of the months ($n$ = 218). $P$ = 0.04.

diagnosed in December were lumped together with those diagnosed in July to September, the $P$-value of survival analysis increased marginally (from 0.0091 to 0.0094).

The difference in the final outcome by the month of the diagnosis could be explained by seasonal hormonal influence on breast cancer, or by changes in social behaviour, such as deferral of medical treatment for holiday for patient or doctor convenience. Seasonal variations have been described in the incidence of a number of noninfectious human disorders, and in the occurrence of breast cancer (Cohen *et al.*, 1983). Melatonin, the major hormone of the pineal gland, has been shown to inhibit the growth of mammary tumours in animal models of human breast cancer, and also the estrogen-responsive human breast cancer cell line MCF-7 in culture in physiological concentrations (Hill & Blask, 1988). On the other hand, melatonin has been reported to increase estrogen receptor binding activity in MCF-7 cells (Danforth *et al.*, 1983). Mason *et al.* (1990) found that women in Auckland, New Zealand, who detected their breast cancer in October to January (spring/summer) between 1976 and 1985 had more favourable prognosis than those who found it during the rest of the year, and the improved survival was found in women aged ⩾50 years with ER and PR positive tumours, and also in women aged <50 years with receptor-negative tumours. Although the natural history of most breast carcinomas is probably long, a change in the hormonal environment could lead to acceleration of tumour growth and an increase in the rate of tumour detection at this time.

However, the evidence in favour of the hormonal hypothesis is not conclusive. The poor prognosis of cancer detected in August to October, and on the other hand in January to February in the 1945–65 series is difficult to explain with hormonal influence. A rapid change in the biological behaviour of breast cancer between November (good prognosis) and December/January (poor prognosis, Table I) is poorly compatible with hormonal influence. If prognosis associated with the months with increasing sunlight (January to June) is compared with the months with decreas-

**Table II** Distribution of prognostic factors by the month of the diagnosis among 738 women with unilateral invasive breast cancer

| | *Diagnosis in 1945 to 1965* | | | | | *Diagnosis in 1980 to 1984* | | | | |
|---|---|---|---|---|---|---|---|---|---|---|
| | *January, February, August, September October* | | *the rest of the year* | | | *July, August, September* | | *the rest of the year* | | |
| *Factor* | *n* | *(%)* | *n* | *(%)* | *P* | *n* | *(%)* | *n* | *(%)* | *P* |
| Tumour necrosis | | | | | | | | | | |
| none | 90 | (59) | 147 | (59) | | 44 | (64) | 204 | (76) | |
| spotty | 24 | (16) | 51 | (21) | | 11 | (16) | 41 | (15) | |
| moderate | 18 | (12) | 36 | (15) | | 14 | (20) | 23 | (9)[a] | |
| extensive | 21 | (14) | 14 | (6) | 0.03 | | | | | 0.02 |
| Mitotic count | | | | | | | | | | |
| rare | 46 | (30) | 101 | (41) | | 29 | (42) | 137 | (51) | |
| >2 | 107 | (70) | 147 | (59) | 0.03 | 40 | (58) | 131 | (49) | 0.18 |
| Tumour size | | | | | | | | | | |
| pT1 (⩽2 cm) | 10 | (7) | 37 | (15) | | 24 | (35) | 112 | (42) | |
| pT2 (2–5 cm) | 88 | (58) | 139 | (56) | | 37 | (54) | 112 | (42) | |
| pT3 (>5 cm) | 25 | (16) | 45 | (18) | | 8 | (12) | 43 | (16)[b] | 0.21 |
| pT4 | 29 | (19) | 27 | (11) | 0.02 | | | | | |
| Age at diagnosis | | | | | 0.67 | | | | | 0.86 |
| Histological type | | | | | 0.79 | | | | | 0.97 |
| Histological grade | | | | | 0.21 | | | | | 0.37 |
| S phase fraction[c] | | | | | 0.41 | | | | | 0.35 |
| Axillary nodal status[d] | | | | | 0.23 | | | | | 0.39 |
| Distant metastases at diagnosis (M1) | | | | | 0.95 | | | | | 0.42 |
| Estrogen receptor content[e] | | | | | – | | | | | 0.46 |
| Progesterone receptor content[f] | | | | | – | | | | | 0.09 |

[a]Moderate and extensive combined. [b]pT3 and pT4 combined. [c]The number of cases above and below the median value were compared. [d]Post-surgical (pN) data used. $n$ = 316 in 1945–65, and $n$ = 296 in 1980–84. [e]No cut-off value produced $P$ <0.2. Cut-off value 10 fmol mg$^{-1}$ protein shown, $n$ = 263. [f]The cut-off value producing the smallest $P$-value shown (25 fmol mg$^{-1}$ protein, $n$ = 238, 52% of PR values low in July to September *vs* 38% during the rest of the year).

ing light in either of the two series, no difference is found ($P = 0.51$ in the 1980–84 series, and 0.95 in the 1945–65 series). Furthermore, there was no difference in mortality from breast cancer in either series if any 6-month period was compared with the rest of the year.

The number of diagnosed cases was usually less than the median during the months associated with unfavourable prognosis. If the months with less than the median of diagnosed cases are tested against the months with cases more than the median, the months with more diagnoses are associated with better survival in the 1980–84 series ($P = 0.05$), but not in the 1945–65 series ($P = 0.26$). The smaller number of cancers detected during the months associated with greater mortality, and the finding of more cancers with adverse prognostic factors, with a large primary tumour size, a high mitotic count, and extensive tumour necrosis (Table II), might suggest that women with less aggressive cancers may postpone their contact with the medical personnel longer, and do not seek for treatment during the summer holidays or at the Christmas time, which are popular holiday periods. However, we find no significant difference in the duration of symptoms or signs caused by breast cancer preceding the diagnosis between the unfavourable months and the rest of the year in either series, nor between June to September and October to December in the 1980–84 series.

In the Auckland series (Mason *et al.*, 1990) the months associated with longer survival were October to January, which corresponds to April to July in the Northern Hemisphere. Unlike in the present series the month of initial tumour detection was primarily recorded in the New Zealand series, and their median delay to histological diagnosis was about 2 months, and the mean delay about 3 months (Neave *et al.*, 1990, Holdaway *et al.*, 1990). If the time lag between tumour detection and diagnosis is assumed to be from 2 to 3 months, according to Auckland data cancers diagnosed histologically in June to September or in July to October in the Northern Hemisphere should be associated with favourable prognosis. In the Turku data the opposite is found, cancers diagnosed in June to September or in July to October have inferior prognosis ($P = 0.02$ and 0.06, respectively, in the 1980–84 series). In the New Zealand data the lowest mean monthly breast tumour progesterone receptor concentrations were found in samples taken in August and September (late winter, Holdaway *et al.*, 1990). These apparently contradictory results may not, however, refute the hormonal hypothesis, because racial factors may be of importance, and the seasonal changes in lightness are considerably greater in Turku (latitude 60°) than in Auckland (latitude 37°).

Some breast cancers are found incidentally by the medical personnel or in screening, and in their detection seasonal hormonal factors are likely to have less importance. The frequency of such cancers is known only for our 1980–84 series, where 38 breast carcinomas were found when the breasts were investigated by a nurse in conjunction with screening for uterine cervical cancer, and further 49 cancers were incidentally found by medical personnel. If the screen-detected or both screen-detected and the incidentally found cancers are removed from the survival analysis, cancers diagnosed in July to September still have inferior prognosis ($P = 0.008$ and 0.047, respectively), and less cancers than the median are still diagnosed in July, August and September.

In accordance with the present data, Jacobson and Janerich (1977) found using the New York State Cancer Registry tumour ER content to be ten times higher in May than in September, May to have the highest frequency of tumour diagnosis, and August, September and December to have the lowest. On the other hand, Hrushesky *et al.* (1979) from Minneapolis found ER content to be higher in late autumn than in spring, the month with the highest concentrations was November.

In summary, prognosis of breast cancer was dependent on the month of diagnosis, cancers diagnosed in July to September had poorer prognosis. In a multivariate analysis the season of breast cancer diagnosis was not an independent prognostic factor. The reasons for the altering prognosis by the month of the diagnosis remain speculative, but more cancers with less adverse prognostic features were detected at other times of the year.

### References

COHEN, P., WAX, Y. & MODAN, B. (1983). Seasonality in the occurrence of breast cancer. *Cancer Res.*, **43**, 892.

COX, D.R. (1972). Regression models and life-tables. *J. R. Stat. Soc.*, **34** (B), 187.

DANFORTH, D.N., TAMARKIN, L. & LIPPMAN, M.E. (1983). Melatonin increases oestrogen receptor binding activity of human breast cancer cells. *Nature*, **305**, 323.

HILL, S.M. & BLASK, D.E. (1988). Effects of the pineal hormone melatonin on the proliferation and morphological characteristics of human breast cancer cells (MCF-7) in culture. *Cancer Res.*, **48**, 6121.

HOLDAWAY, I.M., MASON, B.H., MARSHALL, R.J., NEAVE, L.M. & KAY, R.G. (1990). Seasonal change in the concentration of progesterone receptor in breast cancer. *Cancer Res.*, **50**, 5883.

HRUSHESKY, W., TESLOW, T., HALBERG, F., KIANG, D. & KENNEDY, B.J. (1979). Temporal components of predictable variability among the 1-year scale in estrogen receptor concentration of primary human breast cancer. *Proc. Am. Assoc. Cancer Res.*, **20**, 331.

JACOBSON, H.I. & JANERICH, D.T. (1977). Seasonal variation in the diagnosis of breast cancer. *Proc. Amer. Cancer Res.*, **18**, 93.

MASON, B.H., HOLDAWAY, I.M., STEWARD, A.W., NEAVE, L.M. & KAY, R.G. (1990). Season of initial discovery of tumour as an independent variable predicting survival in breast cancer. *Br. J. Cancer*, **61**, 137.

NEAVE, L.M., MASON, B.H. & KAY, R.G. (1990). Does delay in diagnosis of breast cancer affect survival? *Breast Cancer Res. Treat.*, **15**, 103.

TOIKKANEN, S., JOENSUU, H. & KLEMI, P. (1989). The prognostic significance of nuclear DNA content in invasive breast cancer – A study with long-term follow-up. *Br. J. Cancer*, **60**, 693.